# Robust Permutation Test of Intraclass Correlation Coefficient for Assessing Agreement

**DOI:** 10.3390/cancers17162713

**Published:** 2025-08-21

**Authors:** Mengyu Fang, Alan David Hutson, Han Yu

**Affiliations:** Department of Biostatistics and Bioinformatics, Roswell Park Comprehensive Cancer Center, Buffalo, NY 14263, USA; mengyu.fang@roswellpark.org (M.F.); alan.hutson@roswellpark.org (A.D.H.)

**Keywords:** ICC, agreement, permutation test

## Abstract

**Background:** Inter-rater reliability is critical in oncology to ensure consistent and reliable measurements across raters and methods, such as when evaluating biomarker levels in different laboratories or comparing tumor size assessments by radiation oncologists during therapy planning. This consistency is essential for informed decision-making in both clinical and research contexts, and the intraclass correlation coefficient (ICC) is a widely recommended statistic for assessing agreement. This work focuses on hypothesis testing of the ICC(2,1) with two raters. **Methods:** We evaluated the performance of a naive permutation test for testing the hypothesis $H_{0}:\mathrm{ICC}=0$ and found that it fails to reliably control the type I error rate. To address this, we developed a robust permutation test based on a studentized statistic, which we prove to be asymptotically valid even when paired variables are uncorrelated but dependent. **Results:** Simulation studies demonstrate that the proposed test consistently maintains type I error control, even with small sample sizes, outperforming the naive approach across various data-generating scenarios. **Conclusions:** The proposed studentized permutation test for ICC(2,1) offers a statistically valid and robust method for assessing inter-rater reliability and demonstrates practical utility when applied to two real-world oncology datasets.

## Simple Summary

When different people assess the same medical image or patient test, it is important that their results agree to ensure accurate diagnoses and research findings. A common way to measure this agreement is with a statistic called the intraclass correlation coefficient. However, traditional methods for testing it rely on strong assumptions about the data, which often do not hold in real-world settings. This can lead to unreliable conclusions. We developed a new method that works even when the data is irregular or limited, using a technique called permutation testing. Through computer simulations and real medical examples, we show that our method provides more accurate and consistent results than standard approaches. This helps researchers and healthcare professionals better judge the quality of measurements, leading to more reliable science and clinical decisions.

## 1. Introduction

The Intraclass Correlation Coefficient (ICC) is an important statistical measure for assessing the level of agreement or consistency between two or more continuous variables, frequently used in disciplines such as psychology, medicine, and social sciences [1,2,3,4,5,6]. It is commonly used for evaluating the reliability of measurements across different raters, instruments, or repeated assessments. By quantifying the proportion of variability in data attributable to the variables of interest rather than measurement error, ICC offers a means of evaluating the quality and consistency of data collection methods. Hypothesis testing on ICC is an important step for the conducting inference on this agreement statistic.

In cancer studies, the role of ICC is particularly critical due to the complex and often multi-center nature of clinical trials and diagnostic assessments [7,8,9]. Reliable and consistent measurement of tumor size, biomarker levels, or imaging interpretations across different observers or institutions is essential for ensuring valid comparisons and reproducibility of results [10,11]. High ICC values in such contexts confirm that observed variations are due to true biological differences rather than inconsistencies in measurement, thereby strengthening the integrity of research findings and supporting robust clinical decision-making. Compared to Lin’s concordance correlation coefficient (CCC) [12], another commonly used measure of agreements which applies to two fixed raters, the ICC can be generalized to scenarios with randomly selected raters.

The commonly used test for ICC usually relies on normality assumptions, but often suffers from poorly controlled type I error when the assumption does not hold. A permutation test is often considered exact for testing zero Pearson and Spearman correlation coefficients, and this is a promising alternative for hypothesis testing of ICC as well. However, recent work has shown that such tests are not exact for testing zero correlation coefficients when the data does not follow the bivariate normal distributions, and a studentization of the test statistic can make the test asymptotically exact [13,14,15,16]. Further, Hutson and Yu showed that when the two variables do not follow bivariate normal distributions, a naive permutation test CCC generally does not control the type I error rate at the desired level [14]. Therefore, the question is whether a similar issue exits for ICC.

The inferences about ICC have been extensively studied [17,18,19,20]. However, the standard methods, such as the *F* test, often assume that data follow a bivariate normal distribution, a condition that is frequently violated in real-world datasets. When data deviate from this assumption, such as in the presence of skewness, heavy tails, or outliers, traditional ICC methods can yield inaccurate or misleading results, undermining the reliability of conclusions drawn from the data. To address these challenges, this paper focuses on the ICC(2,1) with two raters, which is under two-way random model for assessing absolute agreements, and introduces a new testing procedure. Our proposed test can better account for the complexities of non-normal data distributions, allowing for more accurate and reliable agreement assessments. The goal of this paper is to present this novel test and illustrate its advantages through simulations and real-world examples. We believe that our method represents a significant advancement in the assessment of agreement between continuous variables, offering a more reliable approach for complex, non-normal data.

## 2. Intraclass Correlation Coefficient and Measurement of Agreement

ICC is a family of reliability indices used to assess the consistency or agreement of measurements made on units that are organized into groups. Unlike the Pearson correlation coefficient, which assesses the linear association between two variables, ICC is appropriate when the same quantity is measured multiple times, such as in repeated measurements, rater evaluations, or test–retest designs.

ICC models are derived from the analysis of variance (ANOVA) framework and can differ based on three main dimensions: the type of effects model (one-way vs. two-way; random vs. mixed), the unit of measurement (single vs. average), and the type of agreement being assessed (consistency vs. absolute agreement). Among different types of ICC, we focus on the two-way random effects model, ICC(2,1) with two raters, where all subjects are rated by the same set of raters randomly selected from a larger population.

Specifically, two-way random effects model with *n* subjects and *k* raters, where $x_{ij}$ is the rating for subject *i* by rater *j*:

$$x_{ij}=\mu +s_{i}+r_{j}+e_{ij}$$

where μ is the overall mean, $s_{i}∼\left(0,\sigma_{s}^{2}\right)$, $r_{j}∼\left(0,\sigma_{r}^{2}\right)$, and $e_{ij}∼\left(0,\sigma_{e}^{2}\right)$.

This model partitions the total variance into components due to subjects, raters, and residual error. Further, we define MSB as the mean square between subjects, MSR as the mean square for raters, MSW as the residual mean square, k=2 is the number of raters, and *n* is the number of subjects. Thus, we have

$$E\left(MSB\right)=\sigma_{e}^{2}+k\sigma_{s}^{2},$$

$$E\left(MSW\right)=\sigma_{e}^{2},$$

$$E\left(MSR\right)=\sigma_{e}^{2}+n\sigma_{r}^{2}.$$

The single-measure, absolute agreement version, ICC(2,1), is given by [21]:

(1) $$\mathrm{ICC}\left(2,1\right)=\frac{MSB-MSW}{MSB+\left(k-1\right)MSW+\frac{k}{n}\left(MSR-MSW\right)}.$$

## 3. Permutation Test of Intraclass Correlation Coefficient of Agreement with Two Raters

The intraclass correlation coefficient ICC(2,1) is typically evaluated under normality assumptions, which often perform poorly when normality assumptions are violated. In such cases, permutation tests are considered a robust alternative. However, Romano and DiCiccio have demonstrated that a naive permutation test for Pearson’s correlation coefficient fails to adequately control the type I error rate under non-normality due to violations of the exchangeability assumption. This issue can be addressed by using a permutation test based on a studentized statistic. Similar problems have been observed in other measures of agreement and correlation, including CCC [14], Spearman’s correlation coefficient [22], and correlations for ordinal variables [15], where tests based on statistics studentized by the large sample variance can effectively control the type I error rate. Similarly, the large sample variance of ICC(2,1) was given as:

(2) $$V\left(\hat{\rho }\right)=2{\hat{\rho }}^{4}\left[{\left(\frac{1}{\hat{\rho }}-1\right)}^{2}+\frac{n}{k}{\hat{u}}^{2}\right],$$

where $\hat{u}=\frac{k(MSR-MSW)}{n(MSB-MSW)}$[17]. However, this variance estimator tends to be unstable under ρ=0. Studentization by this estimator does not provide a robust test (shown in Appendix B). To address this, we approximate the large sample variance of ICC(2,1) using the variance of Pearson’s correlation coefficient. This approximation is motivated by the observation that when the between rater variation is low and the two raters have similar variance in their ratings. As demonstrated in Appendix A, under these conditions the between-subject and residual variances align with the covariance and variance components of Pearson’s correlation coefficient, making its variance a reasonable surrogate for studentizing ICC. Therefore, we used the large sample variance of Pearson’s correlation for studentization. The detailed procedure for the one-sided test is listed below.

- For *n* pairs of i.i.d. observations $\left(x_{11},x_{12}\right)$, $\left(x_{21},x_{22}\right)$, *…*, $\left(x_{n1},x_{n2}\right)$, estimate the ICC(2,1) as $\hat{\rho }$.
- Estimate the approximated variance by
  $${\hat{\tau }}_{n}^{2}=\frac{{\hat{\mu }}_{22}}{{\hat{\mu }}_{20}{\hat{\mu }}_{02}},$$
  $${\hat{\mu }}_{pq}=\frac{1}{n}\sum_{i=1}^{n}{\left(x_{i1}-{\bar{x}}_{1}\right)}^{p}{\left(x_{i2}-{\bar{x}}_{2}\right)}^{q}.$$
- Calculate the studentized statistic $R=\hat{\rho }/\tau_{n}$.
- Randomly shuffle $\left(x_{12},x_{22},\ldots x_{n2}\right)$ for *B* times. For each permutation, calculate the permuted studentized statistic $R^{k}$, k∈(1,…,B).
- Calculate the *p*-value by
  $$p=\frac{1}{B}\Sigma_{k=1}^{B}I\left(R^{k}>R\right).$$
- Reject $H_{0}$ if p≤α.

## 4. Simulations

We examined Type I error control for the tests introduced above using distributions commonly found in the literature for these examinations in a wide range of settings in DiCiccio (2107) [13]. For our simulation study, we focused on testing $H_{0}:\rho =0$ versus $H_{1}:\rho >0$, with sample sizes n=10,25,50,100,200. Each simulation used Monte Carlo replications 10,000 and the number of permutations used is 1000. We compared the *F* test, Fisher’s *Z*-transformation (Fisher’s *Z* test), naive permutation test (Permute), and studentized permutation test (Stu Permute). The Type I error control for α=0.05 was examined. The specific scenarios are examined as shown below.

1. Multivariate normal (MVN) with mean zero and identity covariance.
2. Exponential given as $\left(X,Y\right)=rS^{T}u$ where $S=diag(\sqrt{2},1)$, r∼exp(1), and *u* are uniformly distributed on the two-dimensional unit circle.
3. Circular given as the uniform distribution on a two-dimensional unit circle.
4. $t_{4.1}$ where X=W+Z and Y=W−Z, where *W* and *Z* are i.i.d. $t_{4.1}$ random variables.
5. Multivariate *t*-distribution (MVT) with 5 degrees of freedom.
6. Mixture of two bivariate normal distributions. Given as $\left(X,Y\right)=WZ_{1}+\left(1-W\right)Z_{2}$ where W∼Bernoulli(0.5), $Z_{1}∼N\left(\left(\begin{array}{c} 0\\ 0 \end{array}\right),\left(\begin{array}{cc} 1 & \rho \\ \rho & 1 \end{array}\right)\right)$, $Z_{2}∼N\left(\left(\begin{array}{c} 0\\ 0 \end{array}\right),\left(\begin{array}{cc} 1 & -\rho \\ -\rho & 1 \end{array}\right)\right)$. We use a range of ρ values: 0.1, 0.3, 0.6 and 0.9 to simulate different degrees of dependency between *X* and *Y* (MVNX_1, MVNX_3, MVNX_6, MVNX_9).
7. Absolute normal distribution (ABNORM). Y=ZX, where *Z* follows standard normal distribution and *X* follows a folded standard normal distribution, thus *Y* has a non-constant variance.
8. Binomial normal distribution (BINORM). X=W+ϵ, where W∼Bernoulli(0.1), $\epsilon ∼N(0,0.05^{2})$, and *Y* is defined to follow a normal distribution with mean 0 and a standard deviation dependent on X+1.
9. Squared normal distribution (SQNORM). X∼N(0,1), and *Y* is a quadratic function of *X* and a standard normal error.
10. Uniform distribution (UNIF). X=W+Z and Y=W−Z, where *W* and *Z* are independent variables following Uniform(−1,1). This represents a scenario of dependency due to contrained support

The results in Table 1 show that all tests control type I errors well under bivariate normal distributions. However, the *F* test, Fisher’s *Z* test, and naive permutation tests tend to be overly conservative for the circular distribution. Meanwhile, they tend to have inflated type I error rates for all other distributions. While for $t_{4.1}$, the type I error is consistently inflated. Note that for these tests, this deviation cannot be corrected as the sample size increases. Instead, they may converge to an arbitrary level, either lower or higher than α.

Fisher’s Z test for the ICC exhibits extremely conservative Type I error rates under non-normal distributions because its variance approximation and normality assumption rely on the data following a bivariate normal distribution in a random effects model. Non-normal data distort the sampling distribution of the ICC, altering the variance and shape of the Z-transformed statistic, which leads to underestimated or inflated standard errors, resulting in poor Type I error rates control. On the other hand, the proposed test consistently controls the type I error rate at desired level across the sample sizes and underlying distributions. Additionally, although the approximation in Appendix A relies on weak rater effects and similar variance between two raters, the test remains valid when these two conditions are not satisfied, such as in ABNORM, BINORM, and SQNORM scenarios.

In addition, we evaluated the power of all test methods under bivariate normal distributions to test $H_{0}:\rho =0$ versus $H_{1}:\rho >0$. The simulation of power was only conducted in bivariate normal distributions because the other tests failed to control type I error in other scenarios; thus, the comparison would not be meaningful. As shown in Table 2, the proposed test performs similarly to the F test and the unstudentized permutation test, with only small differences in power (less than 2%). A slightly larger difference occurs when the sample size is very small (n=10) and the true correlation is strong (ρ=0.6), but this difference quickly disappears as the sample size increases. The proposed studentized permutation test yielded a higher power across all scenarios than Fisher’s *Z* test. These results suggest that the proposed test maintains good power while offering better control of type I error in other settings.

## 5. Real World Examples

### 5.1. Inter-Rater Agreement in CT Radiomics

To demonstrate the practical utility of the proposed studentized permutation test, we first applied it to a computed tomography (CT) radiomics dataset evaluating inter-rater agreement in 19 quantitative imaging features of renal tumors from 106 patients [23]. Two radiologists independently extracted variables including tumor size (volume, long/short axis), attenuation, and various texture features such as entropy, skewness, kurtosis, and uniformity.

We assessed whether the inter-rater ICC for each feature was significantly greater than zero using four methods: the *F* test, Fisher’s *Z*-transformation test, a naive non-studentized permutation test, and our studentized permutation test. For features with high inter-rater agreement, such as tumor volume, attenuation, and entropy, all methods consistently yielded significant results (*p* < 0.05), indicating clear agreement between raters. However, differences among the methods became apparent for features with moderate or borderline ICCs. One illustrative example is the tumor UPP feature, where the *F* test, Fisher’s *Z* test, and naive permutation test produced non-significant *p*-values of 0.175, 0.175, and 0.070, respectively (Table 3). In contrast, the studentized permutation test returned a significant *p*-value of 0.024, suggesting inter-rater agreement that the other methods failed to detect (Table 3). Another example is the kidney entropy feature, which yielded *p*-values of 0.458, 0.458, and 0.252 for the *F*, *Z*, and naive permutation tests, respectively, showing that none of them were statistically significant (Table 3). However, the studentized permutation test produced a strongly significant *p*-value (<0.001), identifying agreement that would otherwise be overlooked (Table 3).

To better understand these discrepancies, we evaluated the distributional assumptions underlying the traditional tests. Specifically, we applied the Shapiro–Wilk test for marginal normality and the Henze–Zirkler test for bivariate normality across all variables that have different test results. The results showed that most features significantly violated normality assumptions, with *p*-values < 0.05 in both tests. An exception was the kidney skewness variable, which did not show a violation in the Henze–Zirkler test (p=0.367). Given these violations, our proposed studentized permutation test is recommended for more reliable inference in such setting because it offers better control of type I error under non-normal and small sample conditions, as supported by the simulation results.

### 5.2. Inter-Rater Reliability for iTUG Test

The use of ICC extends beyond oncology to a wide range of other fields. We further applied our method to data from a clinical study evaluating inter-rater agreement for the instrumented Timed Up and Go (iTUG) test in patients with Parkinson’s disease [24]. The iTUG test captures various movement parameters using wearable sensors, including total iTUG and TUG durations, sit-to-stand (SitSt) and stand-to-sit (StSit) transitions, and their respective flexion and extension phases. Each patient underwent multiple trials on two separate days, assessed independently by two raters. A total of 16 iTUG-derived features were analyzed for inter-rater ICC significance using the same four testing methods.

As shown in Table 4, the results again demonstrate consistent performance for features with high reliability. For example, total TUG duration and StSit durations on both days yielded *p*-values < 0.001 across all four methods, confirming strong inter-rater agreement. However, greater variation among the methods emerged for features with lower ICCs. On Day 1, the iTUG duration yielded non-significant *p*-values using the *F* test (p=0.156), Fisher’s *Z* test (p=0.160), and the naive permutation test (p=0.080) while it identified a statistically significant result using the studentized permutation test (p=0.002), which suggesting that it is more sensitive to moderate agreement even in borderline cases. In addition, for the SitSt extension duration, the *F* test (p=0.038), Fisher *Z* test (p=0.045), and naive permutation test (p=0.026) indicated significance, whereas the studentized permutation test (p=0.096) did not.

A similar pattern was observed on Day 2 for the SitSt and extension durations. While the *F* test, Fisher’s *Z* test, and naive permutation test all returned *p*-values below 0.05, the studentized permutation test yielded more conservative *p*-values of 0.056 and 0.096, respectively. These examples suggest that our method provides better control of type I error, avoiding potentially misleading significance when evidence for agreement is weak.

This interpretation is supported by the original study’s [24] reported ICC values: the inter-rater ICC for iTUG duration on Day 1 was 0.95 (excellent reliability), while the ICCs for SitSt extension duration on Day 1 and Day 2 were 0.56 and 0.57, respectively (moderate reliability). Moreover, results from normality assessments (using Shapiro–Wilk and Henze–Zirkler tests) indicated that most variables deviated from both marginal and bivariate normality, reinforcing the need for methods that do not rely on these assumptions.

Taken together, the iTUG study further confirms the advantages of the studentized permutation test in applied settings. It consistently identifies meaningful inter-rater agreement while mitigating the risks of both inflated and deflated type I error that can arise from the limitations of traditional approaches.

These two real-world examples from CT radiomics and clinical mobility assessment collectively demonstrate the broad applicability and reliability of the studentized permutation test. Its robustness under non-normality, increased sensitivity in borderline cases, and interpretability in small-sample scenarios make it a valuable tool for modern biomedical data analysis, particularly where standard assumptions may not hold.

## 6. Discussion

In this study, we propose a robust concordance correlation permutation test for assessing the null hypothesis of zero ICC(2,1), $H_{0}:\rho =0$. Traditional methods for testing ICC(2,1) rely on normality assumptions, which, as demonstrated in our simulation studies, often result in poorly controlled Type I error rates when these assumptions are violated. While permutation tests offer a nonparametric alternative, previous studies [13,14,15,22] have shown that naive permutation approaches applied to correlation coefficients and the CCC fail to control the Type I error under non-normal conditions, particularly in cases where variables are dependent but uncorrelated.

We show that a similar issue arises when applying naive permutation tests to ICC(2,1). To address this, we develop a permutation test based on a properly studentized test statistic, which maintains accurate Type I error control even with small sample sizes (as few as 10) and under violations of normality. While this study focuses on ICC(2,1) for two raters under a two-way random effects model, the extension to multiple raters is a promising direction for future work. Conceptually, the same studentized permutation framework could be adapted by modifying the test statistic to accommodate the average of multiple ratings per subject. However, the derivation of a suitable variance approximation and the maintenance of exchangeability under permutation become more complex in higher-dimensional settings. Future research is needed to assess the theoretical properties and computational performance of such an extension.

## 7. Conclusions

In conclusion, we developed a robust studentized permutation test for ICC(2,1) that addresses the limitations of traditional parametric and naive permutation methods under non-normal conditions. Through simulations and applied studies, the test demonstrated consistent Type I error control, making it a reliable tool for agreement assessment, especially in biomedical research.

## Figures and Tables

**Table 1:** Type I error rates across distributions and sample sizes.

Distribution	*n*	*F*-Test	Fisher’s *Z* Test	Permute	Stu Permute
MVN	10	0.048	0.036	0.050	0.052
	25	0.051	0.046	0.051	0.051
	50	0.053	0.050	0.052	0.051
	100	0.050	0.049	0.050	0.049
	200	0.052	0.052	0.053	0.054
Exp	10	0.107	0.090	0.114	0.054
	25	0.131	0.124	0.148	0.052
	50	0.145	0.141	0.163	0.054
	100	0.145	0.143	0.160	0.048
	200	0.148	0.147	0.164	0.048
Circular	10	0.012	0.007	0.018	0.054
	25	0.011	0.008	0.011	0.046
	50	0.012	0.010	0.011	0.047
	100	0.011	0.010	0.011	0.047
	200	0.012	0.012	0.012	0.050
*t*_4.1_	10	0.118	0.096	0.103	0.047
	25	0.146	0.137	0.139	0.041
	50	0.162	0.158	0.162	0.040
	100	0.184	0.183	0.184	0.044
	200	0.202	0.200	0.199	0.045
MVT	10	0.072	0.057	0.073	0.046
	25	0.096	0.089	0.096	0.046
	50	0.113	0.109	0.113	0.050
	100	0.113	0.111	0.114	0.047
	200	0.133	0.132	0.132	0.048
MVNX_1	10	0.048	0.034	0.051	0.052
	25	0.050	0.045	0.052	0.049
	50	0.051	0.047	0.050	0.048
	100	0.052	0.050	0.052	0.052
	200	0.053	0.051	0.053	0.049
MVNX_3	10	0.060	0.043	0.048	0.050
	25	0.064	0.057	0.063	0.052
	50	0.066	0.064	0.066	0.052
	100	0.065	0.063	0.052	0.049
	200	0.062	0.062	0.053	0.048
MVNX_6	10	0.094	0.075	0.081	0.049
	25	0.104	0.097	0.101	0.049
	50	0.101	0.098	0.102	0.052
	100	0.105	0.103	0.103	0.051
	200	0.108	0.107	0.107	0.052
MVNX_9	10	0.161	0.139	0.139	0.053
	25	0.159	0.145	0.138	0.050
	50	0.158	0.154	0.153	0.050
	100	0.155	0.154	0.152	0.048
	200	0.161	0.160	0.159	0.050
MVN4_5	10	0.057	0.042	0.049	0.049
	25	0.050	0.046	0.049	0.048
	50	0.052	0.048	0.050	0.050
	100	0.050	0.048	0.048	0.049
	200	0.052	0.051	0.050	0.051
SQNORM	10	0.092	0.053	0.135	0.065
	25	0.124	0.098	0.175	0.060
	50	0.131	0.102	0.182	0.056
	100	0.134	0.107	0.186	0.054
	200	0.141	0.111	0.197	0.050
ABSNORM	10	0.081	0.058	0.107	0.077
	25	0.081	0.063	0.137	0.062
	50	0.085	0.069	0.158	0.066
	100	0.080	0.067	0.160	0.059
	200	0.080	0.069	0.169	0.063
BINORM	10	0.016	0.003	0.093	0.063
	25	0.014	0.010	0.151	0.074
	50	0.017	0.016	0.162	0.070
	100	0.020	0.019	0.167	0.068
	200	0.023	0.022	0.158	0.051
UNIF	10	0.009	0.004	0.090	0.050
	25	0.008	0.005	0.093	0.046
	50	0.007	0.007	0.096	0.045
	100	0.006	0.005	0.056	0.046
	200	0.005	0.005	0.066	0.044

**Table 2:** Power of testing $H_{0}:\rho =0$ versus $H_{1}:\rho >0$ under bivariate normal distribution.

ρ	N	*F* Test	Fisher’s *Z* Test	Permute	Stu Permute
0.2	10	0.131	0.103	0.127	0.121
	25	0.268	0.237	0.257	0.250
	50	0.375	0.399	0.373	0.360
	100	0.651	0.636	0.655	0.637
	200	0.884	0.880	0.883	0.877
0.4	10	0.342	0.271	0.327	0.256
	25	0.654	0.643	0.651	0.601
	50	0.915	0.894	0.911	0.897
	100	0.993	0.994	0.994	0.991
	200	>0.999	>0.999	>0.999	>0.999
0.6	10	0.655	0.561	0.626	0.454
	25	0.953	0.959	0.946	0.922
	50	0.997	0.999	0.999	0.997
	100	>0.999	>0.999	>0.999	>0.999
	200	>0.999	>0.999	>0.999	>0.999

**Table 3:** *p*-values from four inter-rater agreement tests for imaging features. The *p*-values that result in inconsistent conclusions are shown in bold.

Variable	*F* Test	Fish’s *Z* Test	Permute	Stu Permute
Tumor volume	<0.001	<0.001	<0.001	0.012
Tumor longaxis	<0.001	<0.001	<0.001	<0.001
Tumor shortaxis	<0.001	<0.001	<0.001	<0.001
Tumor attenuation	<0.001	<0.001	<0.001	<0.001
Tumor attenuation SD	<0.001	<0.001	<0.001	<0.001
Tumor skewness	<0.001	<0.001	<0.001	<0.001
Tumor kurtosis	<0.001	<0.001	<0.001	<0.001
Tumor entropy	<0.001	<0.001	<0.001	<0.001
Tumor uniformity	<0.001	<0.001	<0.001	0.004
Tumor MPP	<0.001	<0.001	<0.001	<0.001
Tumor UPP	**0.175**	**0.175**	**0.070**	**0.024**
Kidney attenuation	<0.001	<0.001	<0.001	<0.001
Kidney attenuation SD	<0.001	<0.001	<0.001	<0.001
Kidney skewness	**0.036**	**0.037**	**0.046**	**0.066**
Kidney kurtosis	0.365	0.364	0.316	0.350
Kidney entropy	**0.458**	**0.458**	**0.252**	**<0.001**
Kidney uniformity	**0.309**	**0.313**	**0.048**	**0.006**
Kidney MPP	<0.001	<0.001	<0.001	<0.001
Kidney UPP	**0.061**	**0.064**	**0.042**	**<0.001**

**Table 4:** Results of testing $H_{0}:\rho =0$ versus $H_{1}:\rho >0$ for iTUG durations. The *p*-values that result in inconsistent conclusions are shown in bold.

Day	Durations	*F* Test	Fisher’s *Z* Test	Permute	Stu Permute
1	iTUG	**0.156**	**0.160**	**0.080**	**0.002**
	TUG	<0.001	<0.001	<0.001	<0.001
	SitSt	0.002	0.004	0.004	0.002
	SitSt Flex	<0.001	<0.001	<0.001	0.034
	SitSt Ext	**0.038**	**0.045**	**0.026**	**0.096**
	StSit	<0.001	<0.001	<0.001	0.002
	StSit Flex	<0.001	<0.001	<0.001	0.002
	StSit Ext	<0.001	<0.001	<0.001	0.002
2	iTUG	<0.001	<0.001	<0.001	<0.001
	TUG	<0.001	<0.001	<0.001	<0.001
	SitSt	**0.004**	**0.003**	**0.004**	**0.056**
	SitSt Flex	0.284	0.289	0.252	0.228
	SitSt Ext	**0.001**	**0.001**	**0.004**	**0.096**
	StSit	<0.001	<0.001	<0.001	<0.001
	StSit Flex	<0.001	<0.001	<0.001	<0.001
	StSit Ext	<0.001	<0.001	<0.001	0.008

**Figure A1:**
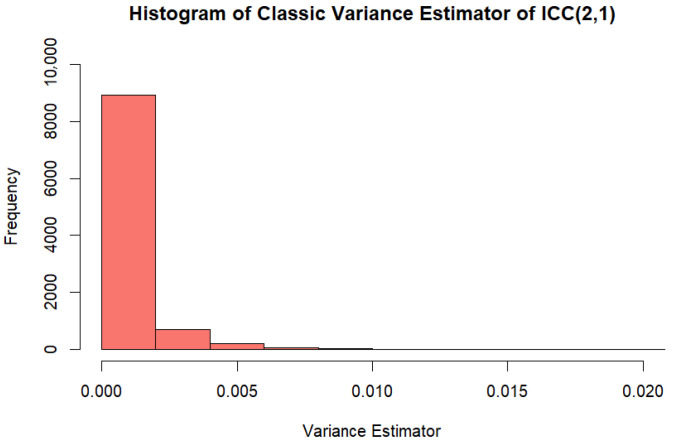
Histogram of Large Sample Variance Estimator of ICC(2,1).

**Figure A2:**
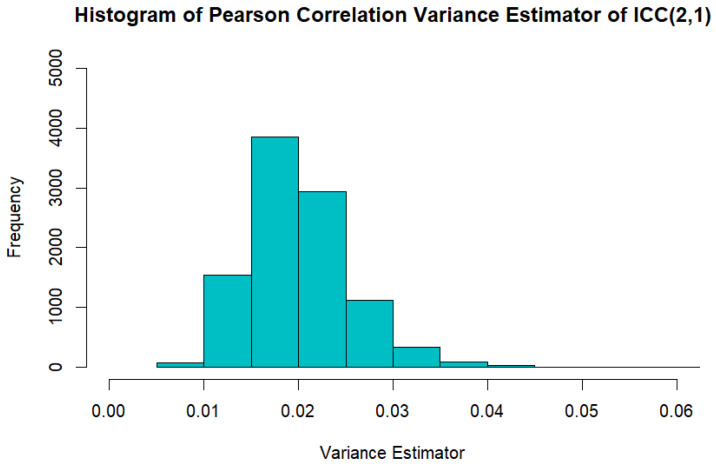
Histogram of Pearson Correlation based Variance Estimator of ICC(2,1).

**Figure A3:**
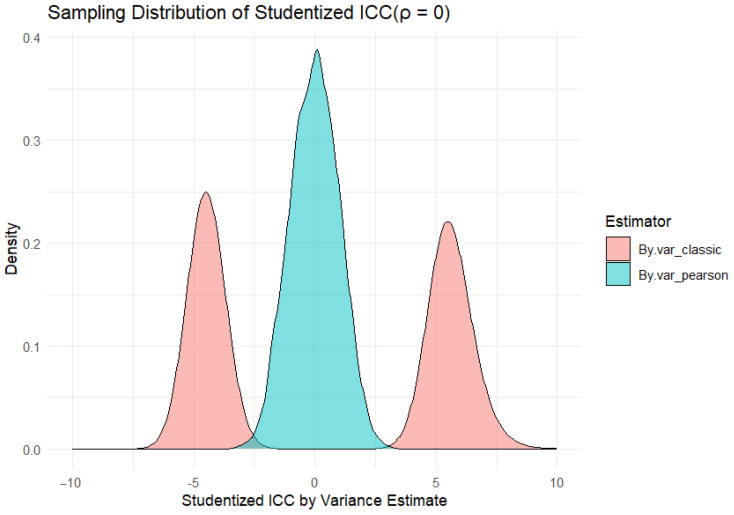
Sampling Distribution of Studentized ICC ρ=0.

## Data Availability

Code is available at https://github.com/hyu-ub/ICC_studentized_permutation_test (accessed on 3 August 2025). The original data presented in the study are openly available in [DANS Data Station Life Sciences] at https://doi.org/10.17026/dans-22j-5w67 (accessed on 19 June 2025) and https://doi.org/10.1371/journal.pone.0195270 (accessed on 19 June 2025).

## Appendix A. Approximation of ICC(2,1) to Pearson Correlation for Two Raters

Here we show that the intraclass correlation coefficient, ICC(2,1), approximates the Pearson correlation coefficient *r* for two raters (k=2) in a two-way random effects model when rater effects are negligible ($\sigma_{r}^{2}\approx 0$). Specifically, we show that MSB−MSW is proportional to $\sum_{i=1}^{n}\left(x_{i1}-{\bar{x}}_{1}\right)\left(x_{i2}-{\bar{x}}_{2}\right)$, and MSB+MSW approximates $\sqrt{\sum_{i=1}^{n}{\left(x_{i1}-{\bar{x}}_{1}\right)}^{2}\sum_{i=1}^{n}{\left(x_{i2}-{\bar{x}}_{2}\right)}^{2}}$.

Consider a two-way random effects model with *n* subjects and k=2 raters, where $x_{ij}$ is the rating for subject *i* by rater *j*:

$$x_{ij}=\mu +s_{i}+r_{j}+e_{ij}$$

where μ is the overall mean, $s_{i}∼\left(0,\sigma_{s}^{2}\right)$, $r_{j}∼\left(0,\sigma_{r}^{2}\right)$, and $e_{ij}∼\left(0,\sigma_{e}^{2}\right)$. For ICC(2,1) with two raters:

$$\mathrm{ICC}\left(2,1\right)=\frac{\mathrm{MSB}-\mathrm{MSW}}{\mathrm{MSB}+\mathrm{MSW}+2(\mathrm{MSR}-\mathrm{MSW})/n}$$

where MSB, MSW, and MSR are the mean squares for subjects, error, and raters, respectively. When $\sigma_{r}^{2}\approx 0$, MSR≈MSW, so

$$\mathrm{ICC}\left(2,1\right)\approx \frac{\mathrm{MSB}-\mathrm{MSW}}{\mathrm{MSB}+\mathrm{MSW}}$$

The Pearson correlation is:

$$r=\frac{\sum_{i=1}^{n}\left(x_{i1}-{\bar{x}}_{1}\right)\left(x_{i2}-{\bar{x}}_{2}\right)}{\sqrt{\sum_{i=1}^{n}{\left(x_{i1}-{\bar{x}}_{1}\right)}^{2}\sum_{i=1}^{n}{\left(x_{i2}-{\bar{x}}_{2}\right)}^{2}}}$$

Firstly, we show the numerator MSB - MSW is proportional to $\sum_{i=1}^{n}\left(x_{i1}-{\bar{x}}_{1}\right)\left(x_{i2}-{\bar{x}}_{2}\right)$.

Define:

$${\bar{x}}_{i}=\frac{x_{i1}+x_{i2}}{2},\;\bar{x}=\frac{1}{2n}\sum_{i=1}^{n}\left(x_{i1}+x_{i2}\right)\approx \frac{{\bar{x}}_{1}+{\bar{x}}_{2}}{2}$$

Assuming negligible rater effects (${\bar{x}}_{1}\approx {\bar{x}}_{2}$):

$${\bar{x}}_{i}-\bar{x}\approx \frac{\left(x_{i1}-{\bar{x}}_{1}\right)+\left(x_{i2}-{\bar{x}}_{2}\right)}{2}$$

$${\left({\bar{x}}_{i}-\bar{x}\right)}^{2}\approx \frac{1}{4}\left[{\left(x_{i1}-{\bar{x}}_{1}\right)}^{2}+2\left(x_{i1}-{\bar{x}}_{1}\right)\left(x_{i2}-{\bar{x}}_{2}\right)+{\left(x_{i2}-{\bar{x}}_{2}\right)}^{2}\right]$$

$$\mathrm{MSB}=\frac{2}{n-1}\sum_{i=1}^{n}{\left({\bar{x}}_{i}-\bar{x}\right)}^{2}\approx \frac{1}{2(n-1)}\left[\sum_{i=1}^{n}{\left(x_{i1}-{\bar{x}}_{1}\right)}^{2}+2\sum_{i=1}^{n}\left(x_{i1}-{\bar{x}}_{1}\right)\left(x_{i2}-{\bar{x}}_{2}\right)+\sum_{i=1}^{n}{\left(x_{i2}-{\bar{x}}_{2}\right)}^{2}\right]$$

$$x_{i1}-{\bar{x}}_{i}=\frac{x_{i1}-x_{i2}}{2},\;x_{i2}-{\bar{x}}_{i}=-\frac{x_{i1}-x_{i2}}{2}$$

$$\mathrm{SSW}=\sum_{i=1}^{n}\left[{\left(\frac{x_{i1}-x_{i2}}{2}\right)}^{2}+{\left(-\frac{x_{i1}-x_{i2}}{2}\right)}^{2}\right]=\sum_{i=1}^{n}\frac{{\left(x_{i1}-x_{i2}\right)}^{2}}{2}$$

$$\mathrm{MSW}=\frac{\mathrm{SSW}}{n-1}=\frac{1}{2(n-1)}\sum_{i=1}^{n}{\left(x_{i1}-x_{i2}\right)}^{2}$$

$${\left(x_{i1}-x_{i2}\right)}^{2}\approx {\left(x_{i1}-{\bar{x}}_{1}-\left(x_{i2}-{\bar{x}}_{2}\right)\right)}^{2}={\left(x_{i1}-{\bar{x}}_{1}\right)}^{2}-2\left(x_{i1}-{\bar{x}}_{1}\right)\left(x_{i2}-{\bar{x}}_{2}\right)+{\left(x_{i2}-{\bar{x}}_{2}\right)}^{2}$$

$$\mathrm{MSW}\approx \frac{1}{2(n-1)}\sum_{i=1}^{n}\left[{\left(x_{i1}-{\bar{x}}_{1}\right)}^{2}-2\left(x_{i1}-{\bar{x}}_{1}\right)\left(x_{i2}-{\bar{x}}_{2}\right)+{\left(x_{i2}-{\bar{x}}_{2}\right)}^{2}\right]$$

$$\mathrm{MSB}-\mathrm{MSW}\approx \frac{1}{2(n-1)}\left[\sum_{i=1}^{n}{\left(x_{i1}-{\bar{x}}_{1}\right)}^{2}+2\sum_{i=1}^{n}\left(x_{i1}-{\bar{x}}_{1}\right)\left(x_{i2}-{\bar{x}}_{2}\right)+\sum_{i=1}^{n}{\left(x_{i2}-{\bar{x}}_{2}\right)}^{2}\right]$$

$$-\frac{1}{2(n-1)}\left[\sum_{i=1}^{n}{\left(x_{i1}-{\bar{x}}_{1}\right)}^{2}-2\sum_{i=1}^{n}\left(x_{i1}-{\bar{x}}_{1}\right)\left(x_{i2}-{\bar{x}}_{2}\right)+\sum_{i=1}^{n}{\left(x_{i2}-{\bar{x}}_{2}\right)}^{2}\right]$$

$$=\frac{1}{2(n-1)}\cdot 2\sum_{i=1}^{n}\left(x_{i1}-{\bar{x}}_{1}\right)\left(x_{i2}-{\bar{x}}_{2}\right)+\frac{1}{2(n-1)}\cdot 2\sum_{i=1}^{n}\left(x_{i1}-{\bar{x}}_{1}\right)\left(x_{i2}-{\bar{x}}_{2}\right)$$

$$=\frac{2}{n-1}\sum_{i=1}^{n}\left(x_{i1}-{\bar{x}}_{1}\right)\left(x_{i2}-{\bar{x}}_{2}\right).$$

Next we derive the approximation of MSB+MSW

$$\mathrm{MSB}+\mathrm{MSW}\approx \frac{1}{2(n-1)}\left[S_{1}^{2}+2\sum_{i=1}^{n}\left(x_{i1}-{\bar{x}}_{1}\right)\left(x_{i2}-{\bar{x}}_{2}\right)+S_{2}^{2}\right]+$$

$$\frac{1}{2(n-1)}\left[S_{1}^{2}-2\sum_{i=1}^{n}\left(x_{i1}-{\bar{x}}_{1}\right)\left(x_{i2}-{\bar{x}}_{2}\right)+S_{2}^{2}\right]$$

where $S_{1}^{2}=\sum_{i=1}^{n}{\left(x_{i1}-{\bar{x}}_{1}\right)}^{2}$, $S_{2}^{2}=\sum_{i=1}^{n}{\left(x_{i2}-{\bar{x}}_{2}\right)}^{2}$.

Combine:

$$=\left(\frac{1}{2(n-1)}+\frac{1}{2(n-1)}\right)\left(S_{1}^{2}+S_{2}^{2}\right)+\left(\frac{1}{n-1}-\frac{1}{n-1}\right)\sum_{i=1}^{n}\left(x_{i1}-{\bar{x}}_{1}\right)\left(x_{i2}-{\bar{x}}_{2}\right)$$

$$=\frac{1}{n-1}\left(S_{1}^{2}+S_{2}^{2}\right)$$

Therefore,

$$\mathrm{MSB}+\mathrm{MSW}\approx \frac{1}{n-1}\left(S_{1}^{2}+S_{2}^{2}\right)$$

When $S_{1}^{2}\approx S_{2}^{2}$, the arithmetic mean approximates the geometric mean:

$$S_{1}^{2}+S_{2}^{2}\approx 2\sqrt{S_{1}^{2}S_{2}^{2}}$$

$$\mathrm{MSB}+\mathrm{MSW}\approx \frac{1}{n-1}\cdot 2\sqrt{S_{1}^{2}S_{2}^{2}}=\frac{2}{n-1}\sqrt{\sum_{i=1}^{n}{\left(x_{i1}-{\bar{x}}_{1}\right)}^{2}\sum_{i=1}^{n}{\left(x_{i2}-{\bar{x}}_{2}\right)}^{2}}$$

Therefore, we have:

$$\mathrm{ICC}\left(2,1\right)\approx \frac{\mathrm{MSB}-\mathrm{MSW}}{\mathrm{MSB}+\mathrm{MSW}}\approx r$$

Thus, the variance can be approximated by:

$$\mathrm{Var}\left(\mathrm{ICC}\left(2,1\right)\right)\approx \frac{1}{n}\cdot \frac{\mu_{22}}{\mu_{20}\cdot \mu_{02}}$$

where $\mu_{22}=E\left[{\left(X_{1}-\mu_{x_{1}}\right)}^{2}{\left(X_{2}-\mu_{x_{2}}\right)}^{2}\right]$, $\mu_{20}=\sigma_{x_{1}}^{2}$, $\mu_{02}=\sigma_{x_{2}}^{2}$.

## Appendix B. Comparison of Large Sample Variance Estimator and Variance Estimator Based on Pearson Correlation When ρ=0

To better demonstrate the instability of the classic large sample variance [17] when ρ=0. We conducted a small numerical simulation to compare the sampling distribution of the classic ICC variance estimator ($var_{classic}$) and the estimator based on the Pearson correlation ($var_{pearson}$) (Appendix A). In this simulation, we focus on the data from multivariate normal distributions with mean zero and identity covariance where the sample size n=50. And this simulation used Monte Carlo replication 10,000.

Figure A1 and Figure A2 display the sampling distributions of two variance estimators for ICC(2,1) under the null hypothesis. The classic variance estimator (Figure A1) is highly skewed and concentrated near zero, indicating poor stability and potential underestimation of variance. In contrast, the Pearson correlation-based estimator (Figure A2) is more symmetric and centered away from zero, suggesting greater consistency and suitability for studentization. Figure A3 displays the sampling distributions of the studentized ICC under ρ=0 using two different variance estimators. The distribution based on the Pearson variance estimator is symmetric and centered around zero, indicating valid standardization under the null. In contrast, the classic variance-based statistic shows a bimodal distribution shifted away from zero, suggesting poor calibration and potential distortion of type I error rates. These results support the use of the Pearson-based variance estimator in the proposed studentized permutation test.
